# Associations between active travel and adiposity in rural India and Bangladesh: a cross-sectional study

**DOI:** 10.1186/s12889-015-2411-0

**Published:** 2015-10-24

**Authors:** Ailsa J. McKay, Anthony A. Laverty, Krithiga Shridhar, Dewan Alam, Amit Dias, Joseph Williams, Christopher Millett, Shah Ebrahim, Preet K. Dhillon

**Affiliations:** Department of Primary Care and Public Health, Imperial College London, London, UK; Centre for Chronic Conditions and Injuries, Public Health Foundation of India, Gurgaon, India; Centre for Global Health Research, Li Ka Shing Knowledge Institute, St Michael’s Hospital, Toronto, Canada; Goa Medical College, Sangath, Goa India; Voluntary Health Services, Chennai, India; Department of Non-communicable Disease Epidemiology, London School of Hygiene and Tropical Medicine, London, UK

**Keywords:** Active commuting, Exercise, Overweight, Obesity, Body mass index, South Asia

## Abstract

**Background:**

Data on use and health benefits of active travel in rural low- and middle- income country settings are sparse. We aimed to examine correlates of active travel, and its association with adiposity, in rural India and Bangladesh.

**Methods:**

Cross sectional study of 2,122 adults (≥18 years) sampled in 2011–13 from two rural sites in India (Goa and Chennai) and one in Bangladesh (Matlab). Logistic regression was used to examine whether ≥150 min/week of active travel was associated with socio-demographic indices, smoking, oil/butter consumption, and additional physical activity. Adjusting for these same factors, associations between active travel and BMI, waist circumference and waist-to-hip ratio were examined using linear and logistic regression.

**Results:**

Forty-six percent of the sample achieved recommended levels of physical activity (≥150 min/week) through active travel alone (range: 33.1 % in Matlab to 54.8 % in Goa). This was more frequent among smokers (adjusted odds ratio 1.36, 95 % confidence interval 1.07–1.72; *p* = 0.011) and those that spent ≥150 min/week in work-based physical activity (OR 1.71, 1.35–2.16; *p* < 0.001), but less frequent among females than males (OR 0.25, 0.20–0.31; *p* < 0.001). In fully adjusted analyses, ≥150 min/week of active travel was associated with lower BMI (adjusted coefficient −0.39 kg/m^2^, −0.77 to −0.02; *p* = 0.037) and a lower likelihood of high waist circumference (OR 0.77, 0.63–0.96; *p* = 0.018) and high waist-to-hip ratio (OR 0.72, 0.58–0.89; *p* = 0.002).

**Conclusions:**

Use of active travel for ≥150 min/week was associated with being male, smoking, and higher levels of work-based physical activity. It was associated with lower BMI, and lower risk of a high waist circumference or high waist-to-hip ratio. Promotion of active travel is an important component of strategies to address the growing prevalence of overweight in rural low- and middle- income country settings.

**Electronic supplementary material:**

The online version of this article (doi:10.1186/s12889-015-2411-0) contains supplementary material, which is available to authorized users.

## Background

Over recent decades, urbanisation and industrialisation have led to declines in physical activity in many countries [1–3]. This has been associated with substantial increases in overweight and obesity, as well as excess cardiovascular- and cancer-related morbidity and mortality [4]. Physical inactivity is now estimated to be the fourth leading cause of death worldwide [4], and a major contributor to non-communicable diseases (NCDs). This is particularly important in low-and-middle-income countries (LMICs), where 80 % of deaths from NCDs occur [5], and urbanisation and industrialisation are increasing in many settings.

In view of the relatively high cardiovascular risk in South Asia [6], physical activity trends in this region, and their associations with cardiovascular risk factors including overweight and obesity, are of interest. The limited data available for India indicate that urban living is associated with reductions in work-based physical activity and increasingly sedentary lifestyles [3, 7]. Trends in rural areas are less clear, but there is some evidence of a reduction in agricultural work and a growing motor vehicle market [8]. India’s large rural population (69 % rural in 2011, compared to 55 % in China and 13 % in Brazil [9]), together with its relatively slow rate of decline in physical activity to date [3], may reflect maintenance of physical activity levels in the rural population.

Active travel (walking, cycling and public transport use) is being promoted as an important component of strategies to increase physical activity levels internationally [5]. This is consistent with a well-developed evidence base from high-income settings demonstrating a protective effect of active travel against overweight/obesity, cardiovascular risk factors and associated mortality [10, 11]. Predictors of active travel in high-income settings include environmental factors (such as low traffic volumes, higher public transport convenience and perceived safety, and a relatively pleasant general environmental aesthetic [12–14]) and individual factors (such as younger age, male sex, urban living, lower distance to work and lower income [13–15]). There is a relative lack of information regarding correlates of physical activity in LMICs [16]. A recent study of middle-income countries, including India, demonstrated associations between higher levels of active travel and male sex, younger age and lower income, in these contexts [17]. This study, additional studies from China [18, 19], and a study using an occupation-based sample from India [20], also indicate that active travel benefits cardiovascular risk factors, including measures of adiposity, in these countries as it does in high-income settings. Data on use and health benefits of active travel in rural LMIC settings specifically are sparse. We conducted the analysis of rural populations in India and Bangladesh reported here, to examine:

1. the socio-demographic correlates of active travel, and
2. associations between active travel and adiposity.

## Methods

### Study design and subjects

Data were obtained from the Chronic Disease Risk Factor Survey, a cross-sectional survey conducted at three rural South Asian sites: Goa and Chennai in India, and Matlab in Bangladesh. Each site was selected on the basis of being a typical rural community in each setting, reflecting the local demographics and containing healthcare facilities to support collection of physical measurements. At each site, households in consecutive village sections, from the healthcare centre outwards, were sampled until households numbered ≥250 and all included sections were fully sampled (*n* = 308 households in Matlab, 309 in Goa, 257 in Chennai). All household residents of 2+ years resident in the area for ≥6 months of the year were invited to participate (*n* = 1143 in Matlab, 1212 in Goa, 940 in Chennai). Participants <18 years, and those for whom physical measurements were not available (see below), were excluded from our analyses.

### Data collection

Data were collected between 06/2011–05/2012 (Matlab), 10/2011–03/2013 (Goa), and 11/2011–03/2013 (Chennai). Trained field-workers (four per site) conducted house-to-house interviews using structured back-translated questionnaires in the local language. Data regarding demographics and chronic disease symptoms, history and risk factors, were collected. Physical activity data were collected using the Global Physical Activity Questionnaire (GPAQ) [21]. Respondents were asked whether they typically undertook ≥10 min episodes of moderate/vigorous physical activity at work or for leisure, and/or for active travel (travel via cycling or walking). The typical duration spent in each type of activity per week was recorded. Participants who completed the questionnaire were invited to attend a field clinic where trained personnel took physical measurements. The measurements included two of standing height (to the nearest 1 mm, using a portable stadiometer (Leicester)), two of weight (to the nearest 100 g, using portable digital scales (Tanita), with participants in light clothing and no shoes), and two of each of waist- and hip-circumference (to the nearest 1 mm, using a taut plastic tape). Measurement equipment was calibrated daily.

### Socio-demographic and additional lifestyle variables

The collected data were used to produced variables describing sex, education (categories: ‘no formal’, ‘any school’, or ‘any higher’ education), smoking status (ever-/never-smoker), moderate/vigorous leisure- and work- related activity (both dichotomised using the 150-min/week cut-off), and oil/butter consumption (categorised as more or less than 1 L/month, based on estimated household consumption, adjusted for number of household residents).

### Active travel variables

A binary active travel variable was used as our main predictor variable. In line with international physical activity guidance [4], participants were dichotomised according to whether they undertook ≥150-minutes of active travel/week. A categorical active travel variable (categories: 0 versus >0 to 150 versus ≥150 min/week) was also produced, in order to consider possible dose-response between the amount of active travel and outcomes. Analyses using this categorical exposure variable were the same as main analyses.

### Adiposity variables

Anthropometric measures were used to generate outcome variables. Body Mass Index (BMI) was calculated using mean height and weight measurements. BMI cut-offs of both ≥23 kg/m^2^ (recommended for Asian populations [22]) and ≥25 kg/m^2^ were used to define overweight and obesity. Obesity (≥30 kg/m^2^) was not considered as an outcome as it was infrequent (*n* = 55, 2.6 %). Mean waist- and hip-circumference measurements were used to calculate waist-to-hip ratios. Binary variables for both waist circumference and waist-hip ratios were produced, using cut-offs recommended for Asian populations (high waist circumference: >85 cm for males, >80 cm for females; high waist-hip ratio: ≥0.9 for males, ≥0.8 for females) [23, 24].

### Statistical analyses

A descriptive summary of study variables was produced for each site. Comparisons between sites were made using chi-square and Kruskal-Wallis tests for categorical and continuous variables (which failed tests of normality and equal variance), respectively. Logistic regression was used to examine associations between ≥150 min/week of active travel and socio-demographic factors as well as lifestyle indices including other modes of physical activity. Linear and logistic regression was used to examine associations between ≥150 min/week of active travel and measures of adiposity. Robust standard errors that allow for correlation within households were used in the regression analyses. Partially adjusted models included age and sex. Fully adjusted models additionally adjusted for site, education, smoking status, oil/butter consumption, and work- and leisure-related physical activity. The analyses examining associations between ≥150 min/week of active travel and adiposity measures were repeated with the categorical active travel variable, and a sensitivity analysis (excluding those ≥65 years; *n* = 166, 7.8 %) was performed. Individuals with missing data were excluded from the regression analyses (*n* = 15 (0.7 %) for analysis of correlates of active travel; *n* = 36 (1.7 %) for analyses of BMI outcomes; *n* = 41 (1.9 %) for analyses of waist circumference and waist hip ratio outcomes).

### Ethics statement

Ethical approval was obtained from the Public Health Foundation of India, Sangath (Goa), Voluntary Health Services (Chennai), the International Centre for Diarrhoeal Disease Research (Matlab), and the Indian Health Ministry (No. 50/5/Indo-CVD/DP/2010-NCD-II). Informed consent was obtained from participants.

## Results

In Goa, Chennai and Matlab, 1143, 1212 and 940 individuals were invited to participate, respectively. Individual level response rates were 92.9 % (Goa), 96.1 % (Chennai) and 96.1 % (Matlab). Non-participation was due to non-consent or no availability after repeated visits. Clinic attendance rates were 99.6 % (Matlab), 85.8 % (Goa) and 97.0 % (Chennai). After excluding individuals <18 years (*n* = 1138) and those that did not attend for clinical measurements (*n* = 145), 2122 participants were included: 740 from Matlab, 734 from Goa, 648 from Chennai. A summary of their socio-demographic characteristics, activity patterns, and adiposity measures are presented in Table 1. Individual components of the socio-demographic/lifestyle data were missing for <0.5 % of participants, components of activity data for <2.0 %, and at least one weight-related outcome for <1.5 %.

**Table 1:** Characteristics of sample

		Matlab	Goa	Chennai	Pooled	p
	n	740	734	648	2122	
Socio-demographic and lifestyle indices						
Age	Mean (SD)	41.3 ± 16.2	40.7 ± 14.3	37.2 ± 14.6	39.9 ± 15.2	<0.001
Sex	% male	40.4	41.4	46.3	42.6	0.064
Education	No formal (%)	27.6	28.1	39.4	31.4	<0.001
	School education (%)	67.5	67.0	53.5	63.1	
	Higher education (%)	4.9	4.9	7.2	5.6	
Ever smoker	%	18.1	85.8	72.7	58.1	<0.001
Oil consumption	≤1 L/month (%)	74.7	95.9	70.7	80.8	<0.001
Physical activity measures						
Active travel	Mean min/week (SD)	235 ± 444	312 ± 445	226 ± 285	258 ± 404	<0.001
	≥150 min/week (%)	33.1	54.8	49.7	45.7	
Work-related physical activity	Mean min/week (SD)	175 ± 581	259 ± 660	1011 ± 1103	460 ± 881	<0.001
	≥150 min/week (%)	15.1	20.7	69.8	33.7	
Leisure-related physical-activity	Mean min/week (SD)	7 ± 60	41 ± 166	52 ± 208	32 ± 155	<0.001
	≥150 min/week (%)	1.6	7.0	9.6	5.9	
Total physical activity	Mean min/week (SD)	419 ± 782	612 ± 815	1285 ± 1164	748 ± 993	<0.001
	≥150 min/week (%)	43.0	63.2	84.6	62.7	
Adiposity outcomes						
BMI (kg/m^2^)	Mean (SD)	21.3 ± 3.6	21.8 ± 4.1	21.2 ± 3.9	21.4 ± 3.9	0.002
	<23	72.6	64.2	70.4	69.0	0.028
	23 - 24.9	12.6	14.8	12.1	13.2	
	25 - 29.9	12.6	17.8	15.1	15.2	
	30 +	2.2	3.2	2.5	2.6	
Waist circumference	>85 cm (males), > 80 cm (females) (%)	26.7	32.3	24.7	28.0	0.004
WHR	≥0.9 (males), ≥ 0.8 (females) (%)	58.5	70.4	59.5	62.9	<0.001

*p*-values refer to differences between sites for each variable

*SD* standard deviation; *BMI* body mass index; *WHR* waist-hip ratio

### Socio-demographic and lifestyle indices

Mean participant age was lower in Chennai than in Goa and Matlab (37.2 versus 40.7 and 41.3 years, respectively; *p* < 0.001). The proportion with no formal education was higher in Chennai than Matlab or Goa (39.4 % versus 27.6 % and 28.1 %; *p* < 0.001). Participants in Matlab were less likely to be ever-smokers than those in Goa and Chennai (18.1 % versus 85.8 % and 72.7 %; *p* < 0.001). Oil/butter consumption was lower in Goa than in Matlab and Chennai (95.9 % consumed ≤1 L/month, versus 74.7 % and 70.7 %; *p* < 0.001).

### Physical activity indices

Forty-six percent of the sample achieved recommended physical activity levels (≥150 min/week) using active travel alone (Table 1). This was less common in Matlab than in Goa and Chennai (33.1 % versus 54.8 % and 49.7 %, respectively; *p* < 0.001). Total physical activity levels were higher in Chennai than in Goa and Matlab (1285 versus 612 and 419 min/week; *p* < 0.001), reflecting higher work-related physical activity levels in Chennai (e.g. 1011 min/week in Chennai, 175 in Matlab). Leisure-related activity was relatively low across all sites: 5.9 % achieved ≥150 min/week.

### Measures of adiposity

Mean BMI was higher in Goa than in Chennai and Matlab (21.8 kg/m^2^ versus 21.2 and 21.3; *p* = 0.002; Table 1). Similar trends were observed for overweight/obese classification (BMI ≥23 kg/m^2^: 35.8 % in Goa, 31.0 % in Chennai, 27.4 % in Matlab; *p* = 0.002), high waist circumference (32.3 % in Goa, 24.7 % in Chennai, 26.7 % in Matlab, *p* = 0.004) and high waist-hip-ratio (70.4 % in Goa, 59.5 % in Chennai, 58.5 % in Matlab; *p* < 0.001).

### Correlates of active travel

Achievement of recommended physical activity levels using active travel (≥150 min/week) was more common in Goa than in Matlab (adjusted odds ratio: 2.13, 95 % CI: 1.59-2.86, *p* < 0.001; Table 2; for Chennai versus Matlab, OR = 1.22, 0.91–1.63, *p* = 0.181). It was also less common among females than males (OR: 0.25, 0.20–0.31, *p* < 0.001). This sex difference persisted when those in the ‘homemaker’ occupational category (*n* = 781/1219 females and 30/903 males) were excluded: OR 0.56, 0.43–0.73, *p* < 0.001 (*a posteriori* test). No association with age was observed (e.g. among those aged 50+ cf. 18–25 years, OR: 0.81, 0.58–1.12, *p* = 0.195). Ever-smokers were more likely to achieve recommended physical activity levels by active travel than never-smokers (OR: 1.36, 1.07–1.72, *p* = 0.011), although this association was only statistically significant in Matlab. Achievement of ≥150 min/week of active travel was more common among individuals who completed ≥150 min/week of work-related physical activity (OR 1.71, 1.35–2.16, *p* < 0.001).

**Table 2:** Associations between socio-demographic and lifestyle indices, and active travel

		Matlab	Goa	Chennai	Pooled
	n	738	729	640	2107
Age group	≥18 to < 25 years	ref	ref	ref	ref
	≥25 to < 35 years	0.74 (0.38, 1.45)	0.58 (0.35, 0.97)*	1.60 (0.97, 2.65)	0.99 (0.72, 1.36)
	≥35 to < 50 years	0.95 (0.53, 1.72)	0.76 (0.44, 1.31)	1.97 (1.14, 3.40)*	1.16 (0.85, 1.59)
	50+ years	0.45 (0.26, 0.81)**	0.59 (0.31, 1.13)	1.08 (0.60, 1.96)	0.81 (0.58, 1.12)
Sex	Male	ref	ref	ref	ref
	Female	0.07 (0.05, 0.12)***	0.48 (0.35, 0.68)***	0.43 (0.31, 0.61)***	0.25 (0.20, 0.31)***
Education	No formal	ref	Ref	ref	ref
	School education	0.97 (0.61, 1.54)	0.73 (0.46, 1.14)	0.74 (0.48, 1.13)	0.69 (0.54, 0.87)**
	Higher education	1.52 (0.60, 3.84)	0.47 (0.20, 1.11)	1.23 (0.53, 2.88)	0.76 (0.48, 1.21)
Ever smoker	No	ref	ref	ref	ref
	Yes	1.70 (1.02, 2.84)*	0.73 (0.47, 1.14)	1.00 (0.67, 1.45)	1.36 (1.07, 1.72)*
Oil consumption	≤1 L/month	ref	ref	ref	ref
	>1 L /month	1.43 (0.89, 2.31)	1.55 (0.76, 3.14)	0.75 (0.50, 1.11)	1.04 (0.81, 1.34)
Leisure-related physical activity	<150 mins/week	ref	ref	ref	ref
	≥150 mins/week	1.60 (0.40, 6.35)	1.69 (0.83, 3.44)	1.12 (0.54, 2.30)	0.96 (0.61, 1.53)
Work-related physical activity	<150 mins/week	ref	ref	ref	ref
	≥150 mins/week	1.31 (0.80, 2.16)	1.60 (1.13, 2.27)**	1.97 (1.33, 2.91)**	1.71 (1.35, 2.16)***
Site	Matlab				ref
	Goa				2.13 (1.59, 2.86)***
	Chennai				1.22 (0.91, 1.63)

Odds ratios for active travel (≥150 min/week) and 95 % confidence intervals are displayed

*p < 0.05, **p < 0.01, ***p < 0.001

### Associations between active travel and adiposity

In unadjusted models, persons achieving ≥150 min/week of active travel had significantly lower BMI and were significantly less likely to be overweight, have a high waist circumference or have a high waist-hip ratio. Findings slightly attenuated in fully adjusted models (Table 3) leaving the overweight finding no longer significant (OR 0.83, 0.68–1.02, *p* = 0.093). The associations between ≥150 min/week of active travel and a lower BMI and lower likelihood of high waist circumference and high waist-hip ratio remained significant in the adjusted models: coefficient for BMI: −0.39 (−0.77 to −0.02; *p* = 0.037), OR for high waist circumference: 0.77 (0.63–0.96; *p* = 0.018), OR for high waist-hip ratio: 0.72 (0.58–0.89; *p* = 0.002). Outcomes of the analyses excluding those >65 years were consistent with these results (see Additional file 1: Table S1).

**Table 3:** Associations between active travel status and weight-related outcomes

Active travel (mins/week)	BMI (kg/m^2^)	BMI ≥ 23 kg/m^2^	BMI ≥ 25 kg/m^2^	High waist circumference	High WHR
	Mean (SD)	%			
<150	21.7 (3.9)	33.5	19.5	31.2	68.1
≥150	21.2 (3.8)	28.4	16.2	24.3	56.9
	Unadjusted coefficient (95 % CI)	Unadjusted OR (95 % CI)			
<150	ref	ref			
≥150	−0.46 (−0.79, −0.12)**	0.79 (0.65, 0.95)*	0.80 (0.63, 1.00)	0.71 (0.58, 0.86)***	0.62 (0.52, 0.74)***
	Partially adjusted coefficient (95 % CI)^a^	Partially adjusted OR (95 % CI)^a^			
<150	ref	ref			
≥150	−0.30 (−0.66, 0.07)	0.89 (0.73, 1.08)	0.91 (0.71, 1.17)	0.81 (0.66, 0.99)*	0.79 (0.65, 0.96)*
	Fully adjusted coefficient (95 % CI)^b^	Fully adjusted OR (95 % CI)^b^			
<150	ref	ref			
≥150	−0.39 (−0.77, −0.02)*	0.83 (0.68, 1.02)	0.84 (0.65, 1.09)	0.77 (0.63, 0.96)*	0.72 (0.58, 0.89)**

High waist circumference: > 85 cm (males), 80 cm (females); high waist-hip ratio (WHR): ≥ 0.9 (males), 0.8 (females)

*BMI* body mass index; *SD* standard deviation; *CI* confidence interval; *OR* odds ratio

*p < 0.05, **p < 0.01, ***p < 0.001

^a^adjusted for age and sex

^b^adjusted for age, sex, site, education, smoking status, oil/butter consumption, and work- and leisure-related physical activity

Outcomes of analyses using the categorical active travel variable were again similar and suggested the duration of active travel was relevant (see Additional file 2: Table S2). There were 677, 476 and 969 participants in the groups reporting no active travel, >0 to <150 min/week and ≥150 min/week, respectively. For the >0 to <150 min/week group, mean weekly duration of active travel was 90.5 min (SD 37.4). For the ≥150 min/week group this was 521.5 min (SD 476.7). In the fully adjusted models, BMI, waist circumference and waist-hip ratio were all observed to trend downwards with increasing levels of active travel. For high waist-hip ratio, differences compared with the group undertaking no active travel were observed for both active travel groups (OR 0.58 (0.41–0.80; *p* = 0.001) in the >0 to <150 min/week group, 0.55 (0.42–0.71; *p* < 0.001) in the ≥150 min/week group). For BMI and high waist circumference outcomes, differences compared with the group undertaking no active travel were observed only for the higher active travel group (BMI: coefficient = −0.28 (−0.84, 0.28; *p* = 0.324) in the >0 to <150 min/week group, −0.54 (−1.00, −0.07; *p* = 0.023) in the ≥150 min/week group; waist circumference: OR = 0.90 (0.66, 1.22; *p* = 0.485) in the >0 to <150 min/week group, 0.73 (0.56,0.96; *p* = 0.027) in the ≥150 min/week group).

## Discussion

### Summary of results

We found that 45.7 % of adults achieved recommended physical activity levels (≥150 min/week) through active travel in rural settings in South Asia. Both males and those that achieved recommended weekly physical activity levels through work-based activity were more likely to achieve this. After adjusting for a variety of socio-demographic factors and other physical activity, ≥150 min/week of active travel was associated with reduced adiposity. Evidence of association was relatively strong for BMI, high waist circumference and high waist-to-hip ratio. Findings using binary BMI-based overweight/obese classifications were consistent, but evidence of association was weaker.

### Correlates of active travel

Few other data regarding active travel are available for South Asian populations. However, in keeping with our observed sex difference in participation in active travel, a recent review demonstrated lower physical activity levels in females, compared with males, in almost all studies of South Asian populations [25]. This review suggested that cultural expectations—including those surrounding care-giving and other domestic activities—likely limit the time available for physical activity among females [25]. Relatively high levels of gender inequality, as demonstrated by Social Institutions and Gender Index (SIGI) scores, in both India and Bangladesh [26], may also be relevant. Country-level analyses have demonstrated an inverse relationship between these scores and active travel among females [27]. Various factors such as monetary costs, safety issues, ethnicity, cultural and religious factors, have been suggested as relevant to this relationship. While the associations between these factors and active travel have been studied in other settings [28–30], as far as we are aware, they have not been studied for South Asian populations, and this area may benefit from future research.

### Active travel and measures of adiposity

The observed associations between active travel and measures of adiposity are in keeping with previous data from India indicative that active travel is associated with a lower risk of overweight/obesity, hypertension and diabetes [17, 20]. The associations are consistent with studies from elsewhere that observed similar associations between active travel, overweight/obesity [11, 18, 31–33], other cardiovascular risk factors [10, 11, 33], cardiovascular disease [10], and related mortality [10]. Additionally, a recent review of observational data from LMICs has demonstrated a role for physical activity more generally, in limiting cardiovascular disease, cancer and all-cause mortality in these settings [34].

### Strengths and limitations of study

This study presents some of the first data on correlates of active travel and associations with adiposity in rural South Asia. The study used representative sampling of populations, with high participation rates, and data collection that allowed for control of important covariates. The main limitation of the study is the use of a cross sectional design, meaning that it remains unclear whether any inference regarding causation is appropriate. The sample size was insufficient for stratified analyses by study site or sex. Physical activity data were based on self-report, and collected via the GPAQ [21]. The GPAQ is similar to the International Physical Activity Questionnaire (IPAQ), which has been associated with overestimation of physical activity levels [35, 36]. Moreover, the particular back-translated version of the GPAQ used here has not been validated. Previous tests of GPAQ reliability and validity versus the IPAQ in a largely LMIC sample showed reasonable correlation between outcomes from the two questionnaires [37]. Both were developed for surveillance studies, and the GPAQ for surveillance in LMICs specifically. There is additional potential for residual confounding as we were unable to account for all factors that may influence both our predictor and outcome measures. For example, ambient temperature and weather conditions, which are likely to have varied across the different sites studied, are important predictors of active travel [27], impact on other forms of physical activity [38], and influence food intake [39, 40]. Additional environmental issues potentially relevant to active travel in South Asian countries include the relatively high levels of road traffic accidents and air pollution [41, 42]. We were unable to consider these, or lung function, all of which may not only influence active travel usage, but also alter the overall benefit-cost ratio relating to active travel in these rural populations.

### Policy implications

Our results suggest that active travel may protect against weight gain in South Asian populations. Although no large-scale evaluation of the costs and benefits of active travel in the South Asian setting has yet been carried out, it appears likely that the overall impact of active travel in rural South Asia would be positive (e.g. [43]). Our data would therefore support the application of current World Health Organization guidelines [5]—which recommend promotion of active travel as a NCD control strategy—in India and Bangladesh. Although current NCD strategies for Bangladesh do encourage environments that facilitate active travel [44], this is less obvious for India [45], and both are non-specific, and lack direct guidance that could help to motivate, as well as facilitate, this behavior. In this regard the lack of data regarding social and environmental impact on active travel in these contexts is concerning, particularly as data from several other contexts suggest that many such factors may have adverse effect on use of active travel. These include road safety concerns, and road infrastructure issues with consequences for traffic density and public transportation, both of which are relatively prevalent in South Asia [46, 47].

Ongoing research on active travel in LMICs is particularly important given the rapidly changing economic, environmental and cultural contexts, including the rapid growth in motor vehicle ownership in many settings. Future research should include a greater emphasis on interventional studies and evaluation of natural experiments to better determine ‘what works’ to increase active travel in LMIC settings. Investigation of the intersection between active travel, social issues such as gender inequality, and potentially relevant environmental factors—including air pollution, road safety, and ambient temperature—would also help shape policy.

## Conclusions

Active travel is a major component of physical activity in rural South Asian populations. It was found to be associated with being male, smoking, and relatively high levels of work-based physical activity. Active travel was associated with more favourable adiposity outcomes. Promotion of active travel is an important component of strategies to address the growing prevalence of overweight in rural LMIC settings.

## Additional files

Additional file 1: Table S1. Associations between active travel status and weight-related outcomes among participants < 65 years. (DOCX 91 kb) Additional file 2: Table S2. Associations between active travel status and weight-related outcomes. (DOCX 105 kb)

## Abbreviations

OR: Odds ratio
BMI: Body mass index
NCD: Non-communicable disease
LMICs: Low- and middle- income countries
GPAQ: Global physical activity questionnaire
CI: Confidence interval
SIGI: Social institutions and gender index
