# Supplementary material for: Associations between active travel and adiposity in rural India and Bangladesh: a cross-sectional study
Source: BMC Public Health. 2015 Oct 24;15:1087. doi: 10.1186/s12889-015-2411-0 (PMC4619428; doi:10.1186/s12889-015-2411-0)
Supplement: Additional file 1: Table S1. — Associations between active travel status and weight-related outcomes among participants < 65 years. (DOCX 91 kb) [file 12889_2015_2411_MOESM1_ESM.docx]

**Supplementary File S1 - Associations between active travel status and weight-related outcomes among participants < 65 years**

High waist circumference: > 85 cm (males), 80 cm (females); high waist-hip ratio (WHR): > 0.9 (males), 0.8 (females)

BMI = body mass index, SD = standard deviation, CI = confidence interval, OR = odds ratio

P<0.05; **p<0.01; ***p<0.001

| **Active travel (min/week)** | **BMI (kg/m^2^)** | **BMI > 23 kg/m^2^** | **BMI > 25 kg/m^2^** | **High waist circumference** | **High WHR** |
| --- | --- | --- | --- | --- | --- |
|  | **Mean (SD)** | **%** | | | |
| **< 150** | 21.9 (3.9) | 34.7 | 20.0 | 32.3 | 68.1 |
| **> 150** | 21.2 (3.8) | 29.0 | 16.8 | 24.4 | 55.8 |
|  | **Unadjusted coefficient (95 % CI)** | **Unadjusted OR (95 % CI)** | | | |
| **< 150** | ref | ref | | | |
| **> 150** | **-0.57 (-0.92, -0.22)**** | **0.77 (0.64, 0.94)**** | 0.81 (0.64, 1.03) | **0.68 (0.55, 0.83)***** | **0.59 (0.49, 0.71)***** |
|  | **Partially adjusted coefficient (95 % CI)** | **Partially adjusted OR (95 % CI)^a^** | | | |
| **< 150** | ref | ref | | | |
| **> 150** | **-0.41 (-0.80, -0.02)*** | 0.85 (0.69, 1.04) | 0.93 (0.72, 1.20) | **0.73 (0.59, 0.91)**** | **0.67 (0.54, 0.84)***** |
|  | **Fully adjusted coefficient (95 % CI)** | **Fully adjusted OR (95 % CI)^b^** | | | |
| **< 150** | ref | ref | | | |
| **> 150** | **-0.49 (-0.88, -0.09)*** | **0.80 (0.65, 0.99)*** | 0.85 (0.66, 1.12) | **0.71 (0.56, 0.88)**** | **0.62 (0.49, 0.77)***** |

^a^adjusted for age and sex

^b^adjusted for age, sex, site, education, smoking status, oil/butter consumption, and work- and leisure-related physical activity
