# Supplementary material for: Associations between active travel and adiposity in rural India and Bangladesh: a cross-sectional study
Source: BMC Public Health. 2015 Oct 24;15:1087. doi: 10.1186/s12889-015-2411-0 (PMC4619428; doi:10.1186/s12889-015-2411-0)
Supplement: Additional file 2: Table S2. — Associations between active travel status and weight-related outcomes. (DOCX 105 kb) [file 12889_2015_2411_MOESM2_ESM.docx]

**Supplementary File S2 - Associations between active travel status and weight-related outcomes**

High waist circumference: > 85 cm (males), 80 cm (females); high waist-hip ratio (WHR): > 0.9 (males), 0.8 (females)

BMI = body mass index, SD = standard deviation, CI = confidence interval, OR = odds ratio

*p<0.05; **p<0.01; ***p<0.001

| **Active travel (min/week)** | **BMI (kg/m^2^)** | **BMI > 23 kg/m^2^** | |  | **BMI > 25 kg/m^2^** | **High waist circumference** | **High WHR** |
| --- | --- | --- | --- | --- | --- | --- | --- |
|  | **Mean (SD)** |  | |  | | **%** |  |
| **0** | 21.7 (3.7) | 32.1 | |  | 17.6 | 31.0 | 70.2 |
| **> 0 to < 150** | 21.7 (4.2) | 35.0 | |  | 21.8 | 31.4 | 65.0 |
| **> 150** | 21.2 (3.8) | 28.3 | |  | 16.0 | 24.3 | 56.9 |
|  | **Unadjusted coefficient (95 % CI)** | **Unadjusted OR (95 % CI)** | | | | | |
| **0** | ref | ref | | | | | |
| **> 0 to < 150** | -0.05 (-0.56, 0.46) | 1.14 (0.88, 1.47) | | 1.31 (0.97, 1.77) | | 1.02 (0.79, 1.32) | 0.79 (0.60, 1.03) |
| **> 150** | **- 0.48 (-0.87, -0.08)*** | 0.83 (0.67, 1.03) | |  | 0.89 (0.69, 1.16) | **0.71 (0.57, 0.90)**** | **0.56 (0.45, 0.69)***** |
|  | **Partially adjusted coefficient (95 % CI)** | **Partially adjusted OR (95 % CI)^a^** | | | | | |
| **0** | ref | ref | | | | | |
| **> 0 to < 150** | -0.02 (-0.53, 0.49) | 1.18 (0.91, 1.54) | **1.36 (1.01, 1.84)*** | | | 1.07 (0.83, 1.40) | 0.90 (0.68, 1.18) |
| **> 150** | -0.30 (-0.72, 0.12) | 0.96 (0.76, 1.20) |  | | 1.05 (0.79, 1.39) | 0.83 (0.66, 1.06) | **0.75 (0.59, 0.96)*** |
|  | **Fully adjusted coefficient (95 % CI)** | **Fully adjusted OR (95 % CI)^b^** | | | | | |
| **0** | ref | ref | | | | | |
| **> 0 to < 150** | -0.28 (-0.84, 0.28) | 0.92 (0.68, 1.24) | | 1.05 (0.75, 1.47) | | 0.90 (0.66, 1.22) | **0.58 (0.41, 0.80)**** |
| **> 150** | **-0.54 (-1.00, -0.07)*** | 0.80 (0.61, 1.04) | |  | 0.86 (0.63, 1.17) | **0.73 (0.56, 0.96)*** | **0.55 (0.42, 0.71)***** |

^a^adjusted for age and sex

^b^adjusted for age, sex, site, education, smoking status, oil/butter consumption, and work- and leisure-related physical activity
